# Development of a Black Caucus within the HIV Prevention Trials Network (HPTN): Representing the Perspectives of Black Men Who Have Sex with Men (MSM)

**DOI:** 10.3390/ijerph17030871

**Published:** 2020-02-03

**Authors:** Christopher Chauncey Watson, Leo Wilton, Jonathan Paul Lucas, Lawrence Bryant, Gregory D. Victorianne, Kerry Aradhya, Sheldon D. Fields, Darrell P. Wheeler, on behalf of the HPTN Black Caucus

**Affiliations:** 1 Gilead Sciences, Foster City, CA 94404, USA; cclwat@gmail.com; 2 Department of Human Development, State University of New York at Binghamton, Binghamton, NY 13902, USA; 3 Faculty of Humanities, University of Johannesburg, Johannesburg 2006, South Africa; 4 Science Facilitation Department, FHI 360, Durham, NC 27701, USA; JLucas@fhi360.org (J.P.L.); karadhya@gmail.com (K.A.); 5 Department of Health Administration, School of Nursing and Health Sciences, Capella University, Minneapolis, MN 55402, USA; oliver2387@bellsouth.net; 6 David Geffen School of Medicine, University of California Los Angeles (UCLA), Los Angeles, CA 90024, USA; gdvvoxx@aol.com; 7 Invitae, San Francisco, CA 93103, USA; 8 Harriet Rothkopf Heilbrunn School of Nursing, Long Island University-Brooklyn, Brooklyn, NY 11201, USA; sheldon.fields40@gmail.com; 9 Iona College, New Rochelle, NY 10801, USA; dwheeler@iona.edu

**Keywords:** Black men who have sex with men (MSM), community-based participatory research, community engagement, qualitative, HIV

## Abstract

Black men who have sex with men (MSM) have disproportionate HIV disease burden in the United States. Black MSM have been underrepresented in biomedical research, including HIV clinical trials, due to a myriad of socio-structural, socio-cultural, and psychosocial factors. The HIV Prevention Trials Network (HPTN) 061, a feasibility study of a multi-component HIV prevention intervention for Black MSM in six US cities, incorporated the development and implementation of a Black Caucus as a culturally grounded model for the integration of Black MSM in clinical trials and research in HPTN. Based on a qualitative methodological approach, we describe the formation and implementation of the Black Caucus from the perspective of Black MSM key community stakeholders. Three major themes emerged from the qualitative narratives: (1) the role of the Black Caucus in shaping the HPTN, (2) how the Black Caucus addresses the needs of Black MSM communities pertaining to the influence of race and sexual identity, and (3) socio-cultural needs of Black MSM. These findings have implications for the provision of culturally congruent expertise, community engagement, cultural mistrust, recruitment and retention of Black MSM in HIV clinical trials, culturally-relevant study design and implementation, and the role of developing Black MSM prevention researchers.

## 1. Introduction

Black men who have sex with men (MSM) have experienced substantial disproportionate HIV infection burden as compared to other populations in the United States (US) [1,2,3,4,5,6]. Prior epidemiologic research has demonstrated that several socio-structural factors have contributed to the disparate HIV infection rates among Black MSM including insufficient access to health care and treatment, higher rates of being un- or under-insured, socio-economic indicators (e.g., poverty, educational inequities, un- or under-employment), housing and food instability, and incarceration [7,8,9,10,11,12,13]. One recent large-scale study (n = 1553) among Black MSM found that economic (recent job loss and financial crisis), legal (recent conviction), and social hardships (unstable housing) were associated with HIV sexual risk behavior and/or a sexually transmitted infection (STI) diagnosis [12]. 

Clinical factors have been shown to affect HIV incidence and prevalence rates among Black MSM, such as infrequent HIV/STI testing patterns, undiagnosed and untreated HIV/STI infections, higher frequencies of HIV/STIs within sexual networks, and suboptimal viral load suppression rates [14,15,16,17,18,19,20,21,22]. In addition, a growing body of research has shown that stigma has influenced the increasing disproportionate HIV disease burden among Black MSM (e.g., racism, homophobia, HIV-related stigma and discrimination, and religiosity-based stigma and discrimination) [8,23,24,25,26,27,28,29,30,31]. Taken together, these studies provide a core basis for understanding key contexts that contribute to the increased HIV vulnerability among Black MSM. 

Black MSM have been under-represented in biomedical research, including HIV clinical trials, due to a constellation of socio-historical, socio-cultural, and psychosocial factors [32]. Research has indicated that Black MSM are less likely to engage in HIV clinical trials, as compared to their White counterparts, due to pervasive cultural mistrust of health care providers and the medical-industrial complex or medical research establishment [25,33,34,35]. Provider bias, past negative experiences with the healthcare system, perceived racist ideologies, and HIV-related conspiracy theories serve as other factors that may impede Black MSM from participating in clinical research studies [12,18,25,28,36,37,38]. Research has also documented the inadequate incorporation of Black researchers in conducting prevention and clinical studies focused on Black communities [39,40,41]. Moreover, for Black MSM who are willing to participate in clinical research studies, barriers to optimal recruitment and retention are common (e.g., inadequate transportation services, insufficient participant incentives, interferences with work commitments, and insufficient capacity to provide culturally-responsive services on the part of study staff can impede recruitment and retention efforts) [37,42,43]. Further, research has indicated that the development of culturally congruent partnerships from the formative phases of the research process often serve as one of the most important factors for the successful recruitment of participants [40]. The inclusion of people of color as key research staff, community leaders, and community members can also help strengthen culturally-relevant research that can be incorporated and sustained in the community [37].

Historically, limited research involving clinical trials has focused on Black MSM in the US [6]. The National Institutes of Health (NIH) funded the HIV Prevention Trials Network (HPTN) to conduct a systematic review and analysis of the available literature and data on the incidence and prevalence of HIV in the US. Based on this review, a domestic agenda for the HPTN was created. The HPTN launched the HPTN 061 protocol entitled “The BROTHERS” (Broadening the Reach of Testing, Health, Education, and Resources & Services) study. The purpose of the study was to determine the feasibility and acceptability of a multi-component HIV prevention intervention for reducing HIV incidence among Black MSM in six US cities: Atlanta, Boston, New York, Los Angeles, San Francisco, and Washington, DC. Due to the importance of this domestic research protocol, a culturally diverse team was constituted in the design and implementation of this study and individuals from the priority population were included as community representatives and investigators. This group of individuals later developed into the HPTN 061 Black Caucus. Here, we report on how we used a qualitative methodological approach to describe how the HPTN 061 Black Caucus was developed and how it provided scientific and community leadership and cultural expertise in the launch and implementation of the HPTN 061 study.

The HPTN 061 Black Caucus was composed of representatives from each of the study’s eight clinical research sites as well as several consultants who were not directly affiliated with a study site. The majority of the members were highly influential in Black MSM communities and successful in their chosen professions, which ranged from research to advocacy. Sites were able to nominate two members of any race, ethnicity, sexual orientation, or gender as long as they possessed relevant knowledge of racial and sexual identities of the men participating in HPTN 061. Based on a series of semi-structured, in-depth interviews with Black MSM key community stakeholders, we developed a better understanding of the importance of the HPTN 061 Black Caucus and its role in the development and implementation of this clinical trial.

### 1.1. The Role of Community-based Participatory Research (CBPR) in Shaping the HPTN

Community-based Participatory Research (CBPR) is a collaborative approach that involves the systematic and equitable integration of the community into the research process [44,45]. CBPR establishes community partnerships that incorporate key community stakeholders and representatives that participate and assume a fundamental role in each phase of the research process (e.g., identifying the research problem, research questions, and relevant research design and data collection methods; conducting the research; and formulating the analysis, interpretation, application, and dissemination of the findings) [46,47,48]. Based on the CBPR framework, one of the primary aims here involved the implementation of the HPTN 061 Black Caucus structure into the HIV Prevention Trials Network as a basis to incorporate the voices of Black MSM and a sustained engagement and commitment to addressing the substantial HIV-related health inequities within Black MSM communities. The utility of CBPR is well demonstrated by the role of the Black Caucus in a multitude of ways: (1) facilitating closer relationships among the researchers, research participants, and broader Black MSM communities; (2) incorporating culturally-relevant theoretical and methodological frameworks; (3) providing critical feedback to funders on ways to strengthen participant recruitment in addition to participant and staff retention; and (3) maintaining cohesion at the clinical research sites.

This multi-tiered partnership between researchers and Black MSM communities is designed to facilitate cooperation, collaboration, and participation during each phase of the research process [49]. An integral component of the CBPR approach is the recognition of the central role of communities, especially historically-underrepresented communities, which have not had sufficient decision-making authority in the research process [44]. Another benefit of CBPR is the fact that its elements build on and utilize resources within the community including community stakeholders [50]. One of the most salient dimensions of CBPR involves its capacity to promote sustained engagement between researchers and participants in addressing persistent inequalities [42]. This fundamental element of the CBPR framework strengthens the promise of facilitating equity and social justice components related to the development and implementation of prevention strategies and improved HIV-related health outcomes for Black MSM communities [51,52]. This is particularly relevant for the HPTN Black Caucus since their input and expertise were instrumental in shaping and informing the research process for HPTN 061. 

### 1.2. History of Black Caucus Development

The HPTN 061 protocol was the first large-scale prospective cohort study among Black MSM in the US that the Division of AIDS within the National Institute of Allergy and Infectious Diseases (NIAID) of the National Institutes of Health (NIH) funded. A protocol team was formed to conceptualize HPTN 061. As the study protocol was evolving, one of the Protocol Co-Chairs sought out selected members of the Black Gay Research Group (BGRG) and the National Black Gay Men’s Advocacy Coalition (NBGMAC) for feedback. Both groups identified significant gaps in the proposed study concept. These limitations were counterintuitive to the implementation of the proposed study. The gaps identified by our interviews can be categorized into three main dimensions: (1) lack of collaboration with Black MSM communities, (2) lack of Black MSM representation in leadership roles, and (3) lack of culturally-relevant data collection methods. 

## 2. Materials and Methods

HPTN 061 was a feasibility study of a multi-component HIV prevention intervention of 1553 Black MSM in six US cities (Atlanta, Boston, Los Angeles, New York City, San Francisco, and Washington, DC). The methods for the study have been described in detail elsewhere [3]. The study was approved by the institutional review boards (IRB) at all participating institutions. Participants were recruited for the study based on community outreach strategies (e.g., community-based organizations, online social networking sites, key informants, and advertising). The inclusion criteria for study participation were as follows: (1) self-identified as Black, African American, Caribbean Black, or multiethnic Black; (2) a man or male at birth; (3) at least 18 years old; (4) reported at least one instance of unprotected anal intercourse (UAI) with a man in the past six months; (5) resided in one of the study’s metropolitan areas and did not plan to move away during the time of study participation; and (6) provided informed consent for the study. 

### 2.1. Sample

For the qualitative study focused on the development and implementation of the HPTN Black Caucus, purposeful sampling was used to select Black MSM community stakeholder interviewees in that we identified persons who were instrumental in designing HPTN 061 and who were involved at the beginning of the formative study dialogue [52]. These interviewees covered areas of public health involving advocacy, research, clinical, and behavioral science. 

### 2.2. Data Collection

Extensive work was conducted to identify interview participants. Meeting minutes for HPTN 061 protocol development calls were reviewed and Black MSM who were pivotal to the design of HPTN 061 were invited to be interviewed. Emails were sent out to potential interviewees and were followed up with telephone calls that delineated what the interview process encompassed. After obtaining informed consent, two trained interviewers conducted semi-structured, in-depth interviews with 11 Black MSM community stakeholders based on an interview guide that was either administered face-to-face at a convenient location identified by the participant or by telephone. The in-depth interviews were approximately 60 minutes in length.

The in-depth interview guide covered three primary areas: (1) introduction and background information, (2) how the Black Caucus addressed the needs of Black MSM, and (3) the function of the Black Caucus. Participants were also asked to discuss their role in HPTN 061 and the impact of the Black Caucus on the design and implementation of HPTN 061. The interviewers asked the participants 12 open-ended questions. Sample interview questions included: How would you describe yourself? What is your connection to the Black MSM community? How did you become involved in the Black Caucus? How do you think HPTN 061 will impact Black MSM communities? What difficulties or challenges have the Black Caucus faced and how did you overcome them? What are the most important contributions that the Black Caucus has made? In-depth interviews were digitally audio-recorded and transcribed verbatim by a professional transcription service. The transcriptions were double-checked for accuracy and completeness. 

### 2.3. Data Analysis

For our analysis, thematic coding was used by the analytic team (CCW, JPL, LW) to analyze the in-depth interviews based on the core principles of grounded theory [53,54]. Based on this method, the analytic team read, reviewed, and independently coded the in-depth interview transcripts using a line-by-line process. The transcripts were analyzed to identify, code, and categorize themes and patterns in the qualitative data. This method provided a context to analyze the structure of the interview and allow relevant themes and patterns to emerge organically from the qualitative narratives. For example, qualitative narratives aimed at extracting text related to the participants’ experiences with the HPTN Black Caucus allowed for sub-themes or codes to be identified, such as the role of cultural responsiveness within the broader theme of socio-cultural needs of Black MSM. The analytic team compared their codes/themes and refined and organized the categories as needed to reach more than 90 percent inter-rater agreement and resolution based on a final coding schema. 

## 3. Results

In this section, we present three main themes related to the formation and implementation of the HPTN Black Caucus that emerged from the qualitative narratives of Black MSM key community stakeholders. For the first theme, we examine the role of the HPTN Black Caucus in shaping the HPTN. The second theme focuses on how the HPTN Black Caucus addresses the needs of Black MSM communities pertaining to the influence of race and sexual identity. The third theme, socio-cultural needs of Black MSM, consisted of two sub-themes: (1) the role of cultural responsiveness and (2) study design and implementation. 

### 3.1. The Role of the Black Caucus in Shaping HPTN

The HPTN Black Caucus was originally composed of representatives from each of the HPTN 061 clinical research locations. Additionally, some representatives who were not affiliated with a site but made significant contributions to the richness of the study protocol were included. Most HPTN Black Caucus members, both present and former, are professional and highly influential in Black MSM communities. The majority of HPTN Black Caucus members function in professional roles, which range from research to advocacy. Many respondents were enthusiastic and very willing to discuss the role of the HPTN Black Caucus in informing HPTN, especially engaging HPTN researchers, about Black MSM communities.

The majority of the respondents articulated that the HPTN Black Caucus developed from interest and critical insights from members of the Black Gay Research Group (BGRG) and the National Black Gay Men’s Advocacy Coalition (NBGMAC). Several salient issues regarding the HPTN emerged as a result of these early conversations. These core issues were: (1) limited inclusion of Black MSM in HPTN 061, (2) an absence of Black researchers in the research process, and (3) the lack of a qualitative component into the HPTN 061 design. One respondent expressed these sentiments most succinctly: 

“We looked at it and we were like, something isn’t right about this. At that time, there was no qualitative piece. So aside from these epidemiological profiles, there was no way to really understand the stories of these Black men.” (Black, Age 50, Community Advocate)

Another respondent echoed this comment and noted:

“So we talked to them, and when the plans were made out for the study, a study that was targeting Black MSM for testing and to get them into treatment, we identified two problems. There was absolutely no qualitative dimension to the study at all at that time. So, when we asked questions such as, how are you going to think about barriers to testing and why people don’t test? What are some of the histories of the relationships between Black people and medicine and why there is skepticism? What are some of the structural issues that prevent or discourage Black MSM from being tested? And what does all this mean when you test people so they can learn their status and get into treatment but there’s no sort of qualitative approach that is designed to actually make this project efficacious?” (Black, Age 45, Social Scientist) 

Almost all of these early members indicated a deep concern that the HPTN 061 protocol lacked a qualitative component. Qualitative research methods capture exactly how Black MSM learn to cope with issues such as homophobia, racism, medical issues, and research staff interaction. It is invaluable in disentangling how immediate and practical problems are dealt with by Black MSM on a daily basis. According to one participant:

“The original draft had no qualitative component to it, and we know that a mixed methodology approach of both qualitative and quantitative measures is probably going to be more helpful when you start [to] research ethnic minority populations. And that’s because of our tradition and our history of passing down oral traditions and we’re talking about researching a group of men who have sex with men who had never been researched at this level, and you’re trying to do this at the community level.” (Black, Age 43, Policy Maker)

Another respondent captured the sentiments of most of the group around the issue of the role of the HPTN Black Caucus in helping shape HPTN 061:

“In terms of the development of the [Black] Caucus, I think there was some feeling at some point that some of the original investigators haven’t had as much depth in dealing with that community, and some wanted to have a [Black] Caucus pulled together to make sure that all of the issues concerning Black gay men were understood and were part of it.” (Black, Age 45, Policy Maker)

The following respondent eloquently captured the perspectives of most of the participants regarding the lack of inclusion of Black gay men’s voices in the research process. This respondent considers this the main problem and powerfully states that finding out why something is the way it is requires more than just a set of numbers. He states: 

“I’m not so much interested in the numbers. And I feel that studies like these are problematic because they don’t want to know why. They just want to know who and how and where. Because why is more complicated. Why is not necessarily easy. There are multitudes of reasons why they are situated in a wide range of contexts. So that was the main problem.” (Black, Age 44, Social Scientist)

These considerations identified the need for the continued integration of race and sexuality components to develop a relevant protocol among Black MSM in the US. As the launch of HPTN 061 came closer, conversations began around convening a formal body that would provide ongoing culturally congruent expertise to the protocol that addressed the current concerns of Black MSM communities. The HPTN Black Caucus formed and began offering the HPTN more internal accountability for the study. 

### 3.2. How the Black Caucus Addresses the Needs of Black MSM Communities (Influence of Race and Sexual Identity)

The qualitative narratives of the early HPTN Black Caucus members reflect a deep concern about race and the lack of inclusion of Black MSM at the planning table and in key decision-making positions and processes. The following two respondents enthusiastically illustrate this point: 

“And, of course, we were like, who do they think is going to do this research? At that point, they were all White researchers. We were like that’s not going to work. And how do they propose to do this going into our communities on this grand scale and they aren’t even including us.” (Black, Age 39, Community Advocate)

“And, then, we started talking with the [current Co-Chairs] about [HPTN] 061, and there were issues that were being raised about the implementation and the [formation] of the investigative team, and were especially concerned about the lack of participation from the Black gay men themselves, including as investigators. And so out of that process, the HPTN leadership committed to forming this Black Caucus that would meet regularly. Because of the advocacy of the two groups [BGRG and NBGMAC] collectively that was used to push the agenda forward with NIH and the protocol co-chair.” (Black, Age 40, Community Advocate)

Two powerful points made by other respondents really made salient the importance of acknowledging the cultural significance of “Blackness”; they assertively noted the following:

“First of all, I think we have to own the fact that this is a race-based study and that it is a study that is focusing on Black men. So at its inception and heart is that we acknowledge this box called Black. And I’m always conflicted by that because I don’t think science has done a good job at saying what that means.” (Black, Age 44, Social Scientist)

“Racial and cultural identity struggles were imminent with developing HPTN 061—addressing these factors became top priority over time and was captured and addressed mostly on behalf of the Black Caucus. It was explicitly clear acknowledgement of such identities in order to press the agenda forward in addressing the public health concern of Black MSM. Due to the salient factors around Black MSM contracting HIV, it was imperative to keep these identities in the forefront while developing the protocol.” (Black, Age 39, Community Advocate) 

Based on these qualitative narratives, the HPTN 061 protocol team began to incorporate suggestions to establish collaboration between the HPTN 061 protocol team, Black Gay Research Group, and the National Black Gay Men’s Advocacy Coalition. The first recommendation that both BGRG and NBGMAC strongly endorsed was that the HPTN 061 research team diversify its leadership. Using its rich networks, the new trilogy not only identified a new co-chair to join the HPTN 061 research team leadership, but also found highly qualified Black MSM and other people of color to work at each of the clinical research sites. These new individuals became official members of the HPTN 061 protocol team.

### 3.3. Addressing the Socio-Cultural Needs of Black MSM

The HPTN formed the HPTN 061 Black Caucus to provide community input, scientific expertise, and support to all aspects of this study. Through a wide range of activities, the HPTN 061 Black Caucus ensured that the design, implementation, analysis, and interpretation of the study were rooted in the appropriate racial and cultural contexts and that the study was responsive to the needs of Black MSM communities. The group also acted as a liaison between the study sites and HPTN 061 leadership, providing a safe venue through which study participants and Black MSM who were working at the study’s clinical research sites could voice their ideas and concerns about the study. Through its work plan, the HPTN 061 Black Caucus was able to raise awareness about many of the socio-cultural nuances of conducting research among Black MSM and within Black MSM communities that helped shape HPTN 061 in several important ways. Our key findings illuminate the contributions of the Black Caucus into two distinct areas: cultural responsiveness and study design and implementation.

### 3.4. The Role of Cultural Responsiveness

One of the themes that emerged in the qualitative narratives related to incorporating the provision of cultural responsiveness into the research process. One participant commented:

“The [Black] Caucus helped the peer health navigators shift from a purely health focus model to a more community-centered focus based on the beginning interaction with the participants. This change recognizes the comprehensive needs that lie within the Black MSM community including housing, employment, and skills development. Working with clinical research sites to establish clinics in convenient locations, ensuring the clinics were open at times that were best for the participants, and establishing a respect continuum for the participants within those settings.” (Black, Age 50, Social Scientist)

Another respondent’s comment illustrates the community benefits of providing cultural responsiveness: 

“Winning over the community’s goodwill. We didn’t have that before. It provided the attention to population issues and culture to ensure that this trial was able to move forward.” (Black, Age 44, Social Scientist)

Many of the respondents noted the importance of having representation at the top level, so the inclusion of a Black gay man as a Principal Investigator (PI) or co-chair was a very important and strategic move. One respondent noted: 

“I think it changed the direction of the study as it was initially developed. I say that because [name of investigator] is a PI now, the qualitative working group, and other things have been developed. You can’t do the study without Black gay men, and I think they realized that.” (Black, Age 39, Community Advocate)

Another respondent’s comment supported this assertion noting that having a Black PI was probably the most important contribution of the Black Caucus, when asked “What are the most important contributions the Black Caucus has made?” He states unequivocally: 

“We certainly think that really pushing to get a Black PI on it. I don’t think that can be underestimated…but this also goes to the larger issue of NIH…. NIH can’t have it both ways. They basically say that they want to support more minority investigators, but they don’t get a lot of applications….So how are Black boys and girls ever going to be inspired to believe that is a welcoming place to exist and to invest themselves in science?” (Black, Age 40, Community Advocate)

### 3.5. Study Design and Implementation

Along with incorporating the qualitative component, the HPTN Black Caucus was instrumental in helping to shape the recruitment and retention methods of HPTN 061. Research suggests that innovative, culturally-relevant methods are more successful than traditional methods for recruiting and retaining members of communities of color in research studies [40,55]. In terms of significance, respondents suggested that including Black MSM voices in the design of the study was of paramount importance. They note the following: 

“You can’t recruit in only traditional ways. We work in social networks using index patients. It’s not necessarily always commonly done, but I also think the way it is done in Black communities is different because it’s not just a Black gay social network.” (Black, Age 39, Community Advocate)

“As a collective, we have brought awareness on how to work and recruit participants in our community. The [Black] Caucus has been able to provide suggestions and recommendations to leadership, and those recommendations have been able to shape the study differently.” (Black, Age 50, Social Scientist)

The wide range of expertise among HPTN Black Caucus members facilitated the building of relationships and sharing of ideas to help push a Black MSM agenda forward. To support the career development of historically-underrepresented researchers, the HPTN Black Caucus endorsed the establishment of the HPTN Scholars Program, which was supported by funding from the National Institute of Allergy and Infectious Diseases (NIAID) [32]. The HPTN Scholars Program funds early-career historically underrepresented investigators to participate in HPTN’s domestic and international research agenda, including HPTN 061 [55]. One of the objectives of the HPTN Scholars program is to provide historically-underrepresented investigators an opportunity to chair or co-chair research protocols and develop quantitative or qualitative components for research conducted by the HPTN Network. The development of the HPTN Scholars Program was based on research that found an inadequate representation of investigators of color who led HIV prevention studies that focus on racially and ethnically diverse populations [39]. 

## 4. Discussion

Our research explored the development and implementation of the HPTN Black Caucus. The purpose of the HPTN Black Caucus was to provide a mechanism for the HPTN leadership to garner increased insight into the specific socio-cultural, socio-structural, and psychosocial complexities of the lived experiences of Black MSM. Three primary themes emerged from the qualitative narratives of Black MSM community stakeholders that related to the formation and implementation of the HPTN Black Caucus. The role of the HPTN Black Caucus in shaping the HPTN was prominent in providing culturally-congruent scientific and community contributions to the richness of the study protocol. The HPTN Black Caucus provided an innovative, interdisciplinary framework for understanding and addressing the needs and concerns of Black MSM communities pertaining to the multi-layered intersections of race and sexual identity. The HPTN Black Caucus served a critical role in facilitating accountability for the incorporation of a cultural responsiveness framework into the study protocol as a basis to facilitate community input, as well as to ensure that the design, implementation, analysis, and interpretation of the study were situated in the racial and cultural contexts of Black MSM communities.

Connected to the role of the HPTN Black Caucus in shaping the HPTN, engaging culturally-responsive members in the protocol development process enriched the HPTN 061 research protocol. The HPTN Black Caucus members strengthened the original HPTN 061 concept in relation to theoretical and methodological rigor as well as cultural responsiveness and relevance for Black MSM communities. Community members served as vital resources for the research investigation related to an incorporation of Black sexuality frameworks involving the socio-historical and socio-cultural contexts of Black MSM communities [56,57]. The development of community-centered relationships with key community stakeholders provided researchers with an opportunity to strengthen their knowledge-base of Black MSM communities in connection to research. This type of cultural connection allows researchers to work in collaboration with communities who have experienced multiple forms of exclusion and marginalization when conducting culturally-grounded community research, as well as addressing the needs of Black MSM based on critical research questions [40,58]. 

Based on the theme of how the HPTN Black Caucus addressed the needs of Black MSM communities, it is imperative to maintain the nexus of race and sexual identity as a conceptual framework in relation to addressing persistent HIV-related inequalities among Black MSM. Having researchers who are aware of the nuances of these socio-cultural frameworks within Black MSM communities became important in developing a protocol that was inclusive of such a diverse population [59,60,61]. Moreover, when conducting research on Black communities, Wyatt et al. [40] indicated that the institutional context of exclusion for Black researchers needs to be considered when integrating culturally-congruent research approaches (e.g., historical, socio-cultural/-structural and personal barriers related to disenfranchisement from the research process). A core component of this work involves the inclusion of researchers that are representative of Black MSM communities and maintain values that are congruent with Black MSM communities [40]. The HPTN Black Caucus was able to provide relevant cultural expertise for overall protocol development and developed a work plan that further enhanced the study in direct ways. Researchers should include key community stakeholders within research protocols at the onset to enhance uptake and cultural responsiveness when developing research protocols.

Cultural responsiveness often had various meanings for HPTN 061 in that it was defined as addressing the socio-cultural contexts of Black MSM culture and implementing a clinical trial within this population as a basis to determine its feasibility. Adapting the model of including culturally responsive staff in clinical trials and prevention research will begin to improve future protocols and ensure acceptability and adaptability within historically-underserved communities [39,41]. These important constituencies will begin to place research in both racial and cultural contexts, provide scientific expertise, and strengthen culturally-relevant theoretical and methodological frameworks. To achieve these objectives, the HPTN Black Caucus was instrumental in providing core leadership with the development of future Black MSM scientific leaders who are equipped with the requisite cultural relevance to meet the needs of Black communities [32]. 

Notably, there were lessons learned about the key struggles or obstacles that were experienced within the HPTN Black Caucus in facilitating a paradigm shift for biomedical and prevention science studies focused on Black MSM communities. First, much of the formative work that was conducted by the HPTN Black Caucus was facilitated without guaranteed funding. Since HPTN Black Caucus members were affiliated with a study site or part of the protocol team, the majority of our work was conducted altruistically in the benefit of Black MSM communities. It would be important for groups who are forming a similar body for historically-underrepresented communities to incorporate a funding component into the design of the study based on relevant resources. Second, as a strategy to develop a research model for groups who are forming a similar body based on a holistic understanding of the life-contexts of historically-underrepresented communities and a commitment to exploring issues of equity and social justice, an integral component would involve articulating a vision for research that incorporates guiding principles (e.g., research needs to be developed, implemented, and applied primarily by researchers reflective of the communities served and in combination with organizations and individuals reflective of those communities that undertake the work of transformative social justice) [62].

Further, based on the considerable efforts that were put forth in the formation of the HPTN Black Caucus, it would be imperative to develop a formative work plan during the initial phase of the process. For example, as a new initiative, substantial engagement occurred between Black MSM researchers/advocates/staff and non-Black protocol team members in that the HPTN was accustomed to a specific framework. In this context, another lesson learned relates to the importance of garnering stakeholder buy-in early within the process. Buy-in coupled with advocacy allowed the HPTN Black Caucus to have a clear runway or path for the development and implementation of programmatic efforts. This advocacy was needed throughout the project to support involvement from managers and senior-level research investigators for the staff members involved. It took proof of concepts (e.g., culturally-informed recruitment and retention strategies) to help shift the mindset of those persons who did not believe that the HPTN Black Caucus was an essential component for a HIV prevention protocol study. Parallel to this process, advocacy from members of the BGRG and NBGMAC was instrumental in providing leadership and accountability to ensure that the needs of Black MSM communities were addressed within a culturally-relevant framework. In this context, the formidable changes that emerged have had a long-lasting positive impact on the HPTN. 

Our findings from this study should be interpreted within the context of a number of limitations. First, the cross-sectional nature of qualitative data collection based on in-depth interviews posed a limitation in that data were captured at one point in time. The use of multiple in-depth interviews may have provided additional insights into the multifaceted complexities of the research process during the full duration of the study. For example, an array of complex domains (e.g., race, sexual identities, and cultural responsiveness) were explored in the study and a longitudinal qualitative design may have allowed for further exploration of these domains. Second, the incorporation of a multi-method approach, using the combination of in-depth interviews and focus groups, may have yielded richer data. Third, based on our CBPR framework, qualitative data collection with members of Black MSM communities reflective of the cities where the study took place may have yielded another layer of community perspectives about the development of the HPTN Black Caucus. Fourth, while we collected standard demographic characteristics about study participants, additional information would have been helpful in understanding the professional interests of the Black MSM community stakeholders who participated in this study. However, this study provided a rich, multi-faceted understanding of an understudied area of clinical research based on the narratives of Black MSM within a culturally relevant framework. 

## 5. Conclusions

Through the provision of cultural expertise in such areas as cultural relevancy, community engagement, participant recruitment, retention, data analysis, and data interpretation, the HPTN Black Caucus provided strong leadership to move the science forward for future research studies involving Black MSM and other marginalized communities [40]. Most research participants and the communities they represent expect that decisions related to the research process in which they are involved reflect the highest level of proficiency, expertise, and understanding regarding their interests. However, historically, this has not always been the case when the research involves Black MSM research participants. The development of the HTPN Black Caucus attempted to fill this void by providing a conduit between the participants and the research leadership team such that participant voices, concerns, experiences, and needs were addressed during each phase of the research process. Through the utilization of a CBPR methodological framework, several conclusions emerged from this study:

1. Black Caucus members were influential and essential in informing the HPTN leadership and research agenda about Black MSM communities and cultural expectations.
2. The Black Caucus strategically and systematically addressed the core needs of Black MSM communities by acting as a liaison between the study sites and HPTN 061 leadership.
3. Black Caucus members eloquently described the conception, evolution, and function of the Black Caucus as a salient component of research involving Black MSM.

Our findings suggest that the development and implementation of the HPTN Black Caucus into the HIV Prevention Trials Network served a critical role in integrating the voices of Black MSM and creating a framework of sustained community engagement, leadership, and commitment to addressing persistent HIV-related inequities within Black MSM communities. For example, standards for community stakeholder engagement changed in HPTN. For any study enrolling a population or using a novel approach, HPTN began incorporating community stakeholder meetings to collect information on the study design. In this context, the HPTN Black Caucus created a guidance document that the HPTN has used when developing efficacy studies to mandate enrollment quotas for studies in the US to ensure adequate representation for Black MSM. 

Our findings suggest that the research model of the HPTN Black Caucus could be utilized as a framework for other historically-underrepresented communities. Elements of the HPTN Black Caucus model have been used in engaging and soliciting cultural expertise from key community stakeholders (e.g., transgender communities) for other HPTN studies in an effort to strengthen study design and community engagement. While the initial iteration of the HPTN Black Caucus focused specifically on HPTN 061, later formulations provided guidance to any research study enrolling or engaging Black MSM and other historically-underrepresented communities. The HPTN Black Caucus developed a cultural responsiveness training that sought to increase understanding of the perspectives of researchers, stakeholders, and research participants from different backgrounds to improve multicultural, multi-racial, -ethnic, -gendered, and -sexuality-oriented clinical research environments. While this training was initially developed to improve rapport with Black MSM participants and community stakeholders, it was later revised to address the needs of transgender people as well. HPTN Black Caucus members identified several representatives from the transgender community to assist in training clinical research sites across biomedical HIV prevention studies. The HPTN Black Caucus has provided ongoing leadership with consultations for HPTN studies with priority populations reflective of historically underrepresented communities.

In the face of what seemed like insurmountable odds, the resilience and tenacious resolve of the HPTN Black Caucus helped communicate to the researchers the benefits of including Black MSM voices in the process. These early warriors challenged and held accountable those persons who would espouse traditional methodologies of conducting research, not only among Black MSM, but in Black communities overall. By insisting that the needs of Black MSM be clearly identified, acknowledged, and supported, the HPTN Black Caucus ushered in a community-based participatory research model that has become the hallmark of the HPTN 061 project and set the standards for future research among this population within all the NIH clinical trial networks. Future research projects should consider the inclusion of priority populations using the tenets of community-based participatory research as a culturally-relevant framework for designing research studies for such populations. Examples like the HPTN Black Caucus can be utilized to ensure that cultural voices are included in research studies which can help to eliminate potential barriers to engaging relevant communities.
